# Impact of YouTube User‐Generated Content on News Dissemination and Youth Information Reception

**DOI:** 10.1111/hex.70408

**Published:** 2025-09-01

**Authors:** Wu Chunqiong, Jiang Shan, Sun Jianhong, Liu Yingqi

**Affiliations:** ^1^ School of Economics and Management Yango University Fuzhou Fujian Province China; ^2^ School of Business Ningbo University Ningbo Zhejiang Province China

**Keywords:** algorithmic influence, digital engagement, media literacy, misinformation, trust in media, user‐generated content, YouTube news

## Abstract

**Background:**

User‐generated content (UGC) on YouTube has reshaped news dissemination, fostered engagement, raised concerns about credibility, algorithmic influence and the spread of misinformation. This study addresses the gap in understanding how UGC engagement, trust and algorithmic awareness influence digital news consumption.

**Methods:**

A convergent parallel mixed‐methods design was employed, integrating survey data (*n* = 100), qualitative interviews and content analysis of 200 YouTube news videos. Data were collected over 6 weeks. Quantitative analyses included ANOVA, multivariate regression and structural equation modelling (SEM), while qualitative data were thematically analysed to contextualise statistical findings.

**Results:**

UGC news consumption (M = 3.21, SD = 1.14) exceeded traditional news (M = 2.95, SD = 1.20), with trust in UGC (M = 3.48, SD = 1.05) surpassing traditional sources (M = 3.12, SD = 1.17). SEM analysis confirmed that UGC engagement significantly increased trust (*β* = 0.42, *p* < 0.001), while algorithmic influence negatively affected trust (*β* = −0.33, *p* = 0.015). Sensationalist content attracted higher engagement (30.0%) but had lower credibility, with misinformation prevalent in 38.0% of analysed videos.

**Conclusion:**

Findings highlight the need for platform transparency, stronger content verification and policy interventions to balance engagement‐driven algorithms and news credibility. Media literacy initiatives are crucial for equipping users with the critical evaluation skills they need.

## 1 Introduction

The evolution of social media platforms has transformed news dissemination, with YouTube emerging as a dominant source, particularly among youth. Traditionally, news production was regulated by professional gatekeepers; however, YouTube has decentralised this process, enabling anyone to generate and circulate news content [1]. This shift is best understood through the lens of uses and gratifications theory (UGT), which explains why younger users turn to user‐generated content (UGC) for authenticity, relatability and participation [2, 3, 4]. Simultaneously, media dependency theory highlights growing reliance on YouTube for multiple informational needs, which amplifies its influence on users' beliefs and attitudes. Unlike traditional outlets, YouTube's algorithmic curation personalises news exposure, reinforcing filter bubbles and restricting content diversity [5, 6, 7, 8]. Algorithmic gatekeeping is critical for examining how recommender systems replace editorial oversight, allowing unverified or misleading content to influence public discourse. Despite this transformation, few studies systematically assess how such algorithmic environments affect young people's ability to evaluate the credibility and trustworthiness of news content.

While UGC's role in health and education has been explored [9, 10, 11, 12], its broader influence on news credibility and engagement remains under‐investigated. Harris et al. [2] noted that YouTubers often blend sincerity with commercial intent, complicating viewer trust. Pérez‐Torres et al. [4] found that UGC fosters identity formation but left questions about its role in news evaluation. Though their focus remained on educational content, Mohamed and Shoufan [13] showed that UGC facilitates learning through comment interaction. Mayrhofer et al. [14] highlighted persuasive UGC tactics that blur the line between journalism and influence, while [15, 16] exposed the dangers of misinformation and the erosion of editorial control. However, most prior research is region‐specific or genre‐limited, often relying on self‐reported data. A comprehensive, cross‐cultural investigation of how UGC affects trust, credibility and news engagement remains lacking, particularly in algorithm‐driven environments.

Recent concerns about misinformation, credibility and algorithmic curation in digital spaces have intensified the scrutiny of UGC, particularly in the context of global health emergencies and sociopolitical disruption. Several studies have documented how false or misleading user‐generated news content circulates more rapidly than verified reports, amplifying risks to informed decision‐making and public trust in institutions. This challenge carries direct public health implications, as misinformation about health, vaccines and emergencies has been shown to influence behaviours, risk perception and compliance with medical guidance. Therefore, enhancing digital media literacy is essential for informed civic participation and protecting collective well‐being.

## 2 Problem Statement

UGC on YouTube has disrupted traditional news channels, enabled the spread of unverified content and raised concerns about credibility, algorithms and misinformation. Lacking editorial oversight, such content influences how young users perceive the trustworthiness of news. Yet, few studies explore how they assess credibility or distinguish fact from fiction in algorithm‐driven environments. This gap limits understanding of UGC's broader impact on digital news trust and literacy.

## 3 Objectives

This study aimed to:

1. Evaluate the impact of UGC on YouTube on young users' trust in news and source credibility.
2. Investigate the impact of algorithmic curation and news‐entertainment convergence on perceptions of news.
3. Evaluate the role of media literacy in enhancing critical evaluation of participatory news content.

## 4 Literature Review

### 4.1 The Rise of UGC and Its Impact on News Consumption

YouTube has transformed youth news consumption through the rise of UGC, replacing traditional gatekeeping by professional journalists with a participatory, decentralised model [17]. While this democratisation enhances inclusivity, it also raises concerns about credibility, misinformation and informational silos [18, 19, 20, 21]. Studies have examined UGC's role in public service broadcasting [15, 22] and demonstrated how broadcasters utilise UGC to enhance real‐time coverage and engagement. However, such findings are context‐specific, given varying media regulations and digital adoption levels globally. Broader cross‐national analyses are required to determine whether UGC's impact is universal or shaped by localised media systems.

Burkey [17] explored millennials' dual roles as news consumers and distributors, identifying YouTube as a central hub for curated news sharing. While this amplifies diverse voices, it simultaneously increases the risk of misinformation. Burkey's generational scope excludes Gen Z and Gen X, whose digital literacy and trust behaviours differ. These differences must be explored to fully understand perceptions of credibility across age groups. Xiang [16] examining participatory journalism in China, found that platforms like Pear Video and Kwai shifted agenda‐setting from media institutions to users, highlighting UGC's democratising potential. However, the regional scope limits generalisability to global platforms like YouTube, which serve broader, culturally diverse audiences. Comparative research across regulatory and cultural settings is needed to understand UGC's role in global news consumption.

### 4.2 Young People's Engagement With UGC and News Consumption

Several studies explore how UGC shapes youth identity, yet its direct impact on news consumption remains underexamined [23, 24, 25, 26]. Pérez‐Torres et al. [4] found that adolescents engage with YouTubers as relatable peers, fostering identity exploration through comment behaviour aligned with UGT, which frames such engagement as socially motivated but not necessarily news‐critical. UGC reinforces gender roles and vocational aspirations among youth, but these studies lacked cross‐cultural generalisability and overlooked news‐literacy implications [13, 19, 27]. In media dependence theories, the increasing reliance on YouTube for identity and information raises vulnerability to misinformation, especially when users struggle to distinguish between entertainment and news. Smith et al. [28] revealed ethical gaps in youth‐oriented vaping content, such as the absence of health warnings and age restrictions, highlighting broader risks associated with unregulated content credibility. These findings underscore the need to assess how adolescents interpret UGC as news, not just as identity content.

### 4.3 Ethical Concerns and Challenges in UGC‐Driven News

Ethical concerns in UGC‐driven news largely stem from the absence of editorial standards [29, 30, 31]. Content creators struggle between clickbait and public‐interest journalism, highlighting how digital platforms blur the lines between news and entertainment, thereby weakening editorial independence [24, 32]. However, this regional scope limits generalisability and overlooks how young users across cultures evaluate UGC credibility. In media dependency theory, cross‐cultural analysis is essential to assess how structural reliance on UGC amplifies misinformation risks [33, 34]. Mayrhofer et al. [14] showed that branded UGC subtly influences young adults more than traditional advertising due to lower persuasion awareness, yet the study neglected how similar framing in news‐related UGC shapes trust.

Studies [27, 35, 36, 37, 38] have emphasised public safety messaging, but did not explore how algorithmic curation on platforms like YouTube filters youth news exposure. This curation impacts news engagement and bias, reinforcing the relevance of algorithmic gatekeeping theory. Meanwhile, dos Santos Catalão [15] proposed a framework for UGC's expressive capacity, but it remains empirically untested in news contexts. To bridge these gaps, this study adopts a unified theoretical framework integrating UGT, MRT and algorithmic gatekeeping to explain how UGC motivates engagement (UGT), how format influences credibility (MRT) and how algorithms shape visibility and trust. This cross‐theoretical model enables a more global and mechanism‐based understanding of youth engagement with UGC news.

## 5 Methods

### 5.1 Research Design

The study used a convergent parallel mixed‐methods design, combining survey data on UGC use, trust and engagement with interviews and focus groups. Triangulation aligned quantitative patterns with qualitative insights to strengthen interpretation.

### 5.2 Participants

The study was conducted at China Southwest Medical University, enrolling participants aged 13–35 from five countries: the US, Brazil, India, China and Japan. Stratified random sampling ensured balanced representation by age, gender, education and location. A power analysis determined the sample size to be 100 (20 per country), with random selection used to minimise bias. Recruitment involved online ads, social media, university mailing lists and direct outreach to content creators. Eligibility required active YouTube use for news and a willingness to join interviews or focus groups. Data saturation was confirmed after three consecutive interviews, during which no new themes emerged. To ensure diversity, 20 additional qualitative participants (4 per country) and 20 content creators were purposively sampled. Incentives were provided to sustain participation.

### 5.3 Ethical Considerations

This study was approved by the Institutional Review Board of China Southwest Medical University (Approval No. SWM‐0098‐DS4). All participants provided written informed consent before participation; for minors aged 13–17, parental permission and participant assent were obtained. The research adhered to the ethical principles of the Declaration of Helsinki, including voluntary participation, data confidentiality and the right to withdraw at any point. Data were anonymised during analysis, and no identifying information was retained.

### 5.4 Survey

A 40‐item questionnaire was developed to assess youth engagement with UGC news, trust in sources and perceived credibility of these sources. It included five sections: Demographics (6 items), UGC Engagement (10 items), Trust (8 items), Credibility Assessment (10 items) and Algorithmic Awareness (6 items). Trust and credibility were measured using five‐point Likert scales (1 = Not Trustworthy to 5 = Very Trustworthy), adapted from [39, 40]. Total scores were calculated by summing item responses within each domain. A pilot test (*n* = 30) confirmed internal consistency (α = 0.85 for trust, α = 0.80 for credibility).

### 5.5 Interviews

The qualitative phase consisted of 10 focus groups and 120 semi‐structured interviews, each lasting 45–60 min and guided by a 10‐question protocol, involving 100 youth and 20 creators. Youth interviews covered YouTube news use, trust, credibility strategies and algorithm influence; creator interviews focused on content production, audience engagement and ethical concerns. All sessions followed a structured guide and were transcribed verbatim for thematic analysis across cultural and digital trust models.

### 5.6 Content Analysis of YouTube News Videos

**Figure 1:**
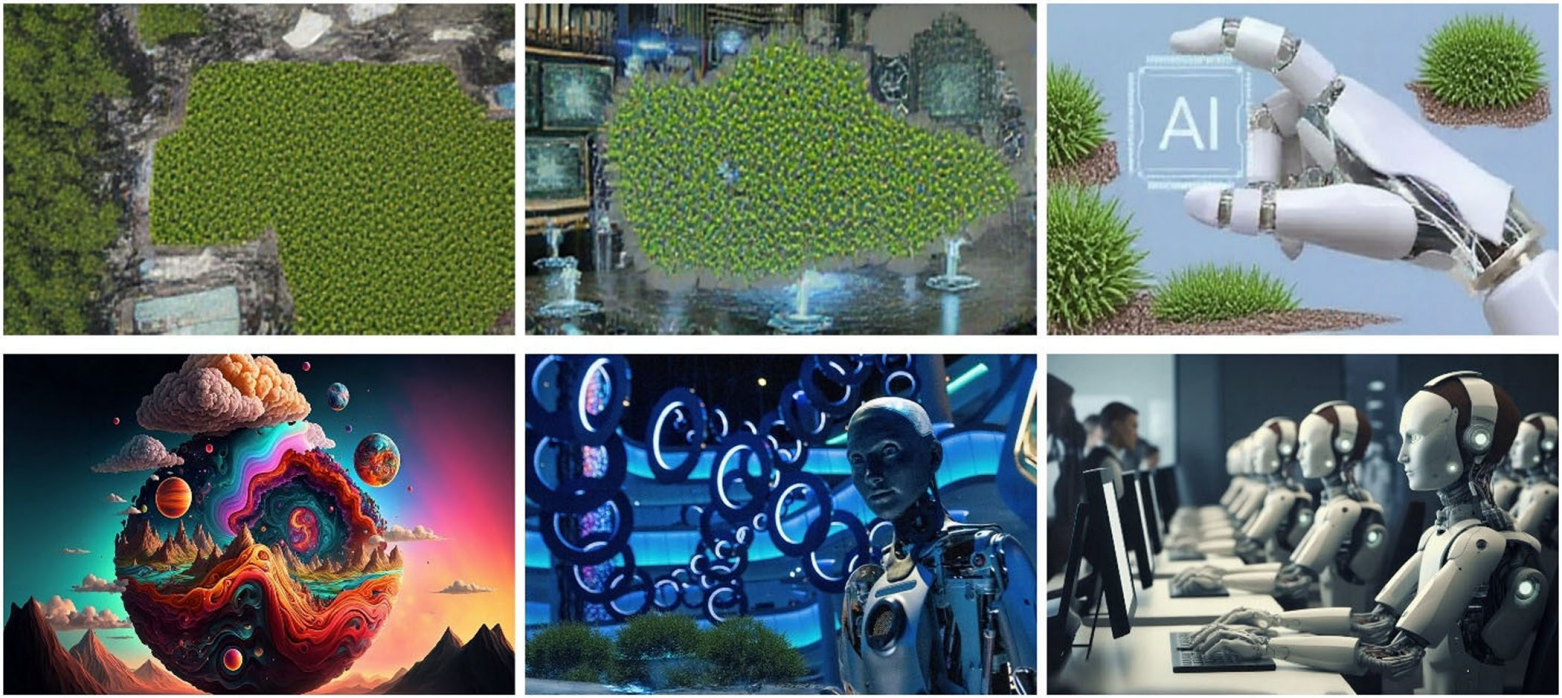
Illustration of YouTube UGC's role in AI‐driven news creation, curation and engagement ethics.

**Figure 2:**
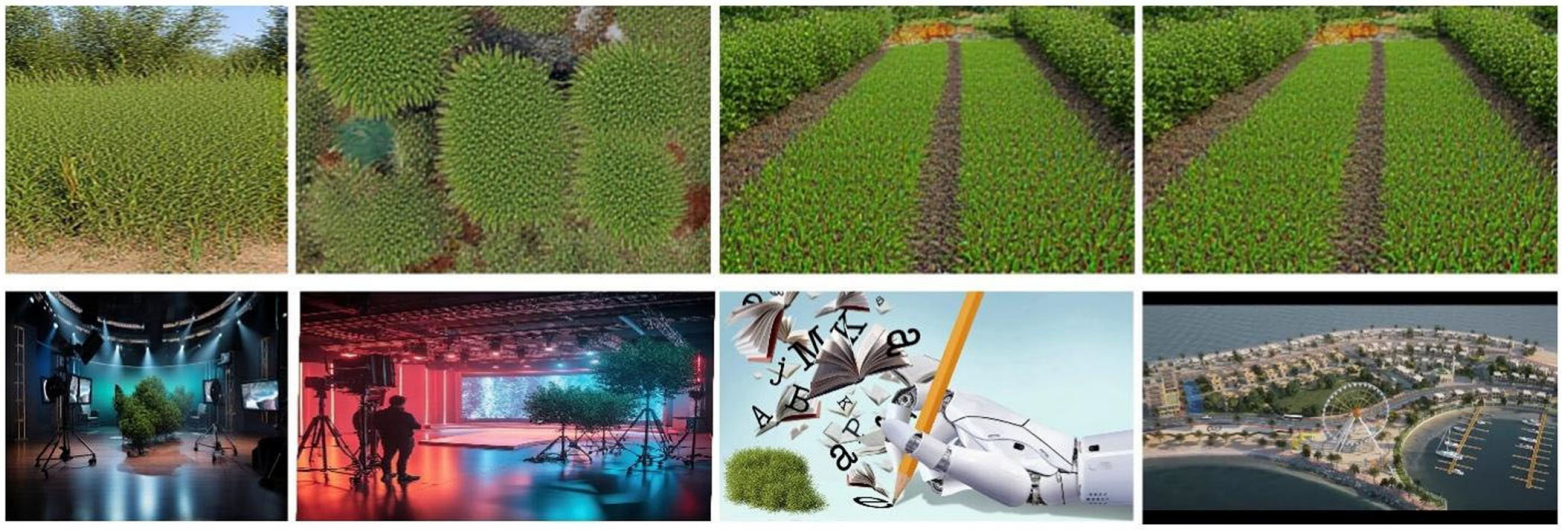
YouTube UGC ecosystem shaped by AI, algorithms, storytelling and audience‐driven news dissemination.

A content analysis of 200 YouTube news videos examined structure, framing and engagement across cultural contexts. Videos were selected based on recent view counts, sharing metrics and topic diversity, including politics, health, entertainment and global affairs. Two coders evaluated 20% of videos (Cohen's kappa = 0.82), resolving discrepancies through consensus. Framing was categorised as sensationalist, neutral, or analytical; credibility was assessed by referencing official data or journalistic sources. Viewer engagement, including likes, dislikes, comments and sentiment, was analysed to gauge patterns of trust and scepticism.(Figure 1, Figure 2).

### 5.7 Statistical Analysis

Quantitative data were analysed using R 4.4.2 (Posit Software, USA) for descriptive and inferential statistics. Descriptive measures included means, medians, standard deviations and frequency distributions. ANOVA and multivariate regression identified group differences and predictors of UGC trust and credibility (*p* < 0.05). Exploratory factor analysis and Structural Equation Modelling (SEM) were conducted using Python 3.9 (Python Software Foundation, USA) to test theoretical associations. Qualitative transcripts were analysed in NVivo 12 (QSR International, Australia) using inductive thematic coding. Two coders ensured reliability (Cohen's kappa), with consensus resolving discrepancies. MAXQDA 2022 (VERBI Software, Germany) supported video content analysis by coding format, framing, sources and engagement metrics. Integrated findings across datasets captured UGC's impact on youth trust and engagement with news.

## 6 Results

**Figure 3:**
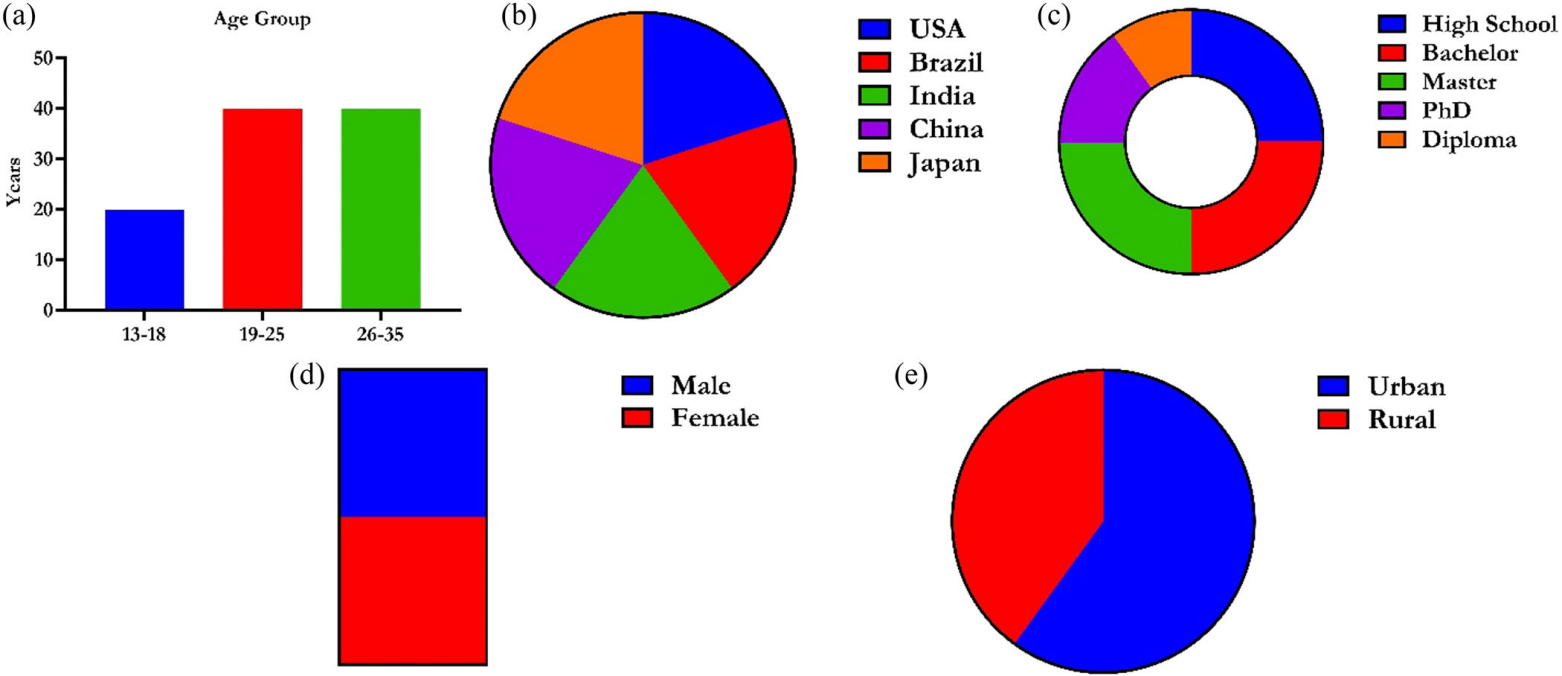
Baseline characteristics of participants based on age group (years), gender, country of origin, education level and location. (a) Age Group, shows the distribution of participants across different age groups (13‐18, 19–25, 26–35). (b) Country of Origin, shows the distribution of participants by their country of origin (U.S., Brazil, India, China, Japan). The label "USA" in the text snippet is a specific example for this category. (c) Education Level, shows the distribution of participants by their highest achieved education level (High School, Bachelor's, Master's, PhD, Diploma). The text "Bachelor Master Diploma" lists some of these categories. (d) Gender, shows the gender distribution of the participants (Male, Female). (e) Geographic Location, shows the distribution of participants based on whether they live in an urban or rural setting.

The study included 100 participants (Figure 3), aged 13–35, with a balanced gender distribution (50% male, 50% female) and equal representation from the U.S., Brazil, India, China and Japan (20% each). Age groups were 13–18 (20%), 19–25 (40%) and 26–35 (40%). Education levels included high school (25%), bachelor's (25%), master's (25%), PhD (15%) and diploma (10%). Geographically, 60% of the population lived in urban areas and 40% in rural settings.

**Figure 4:**
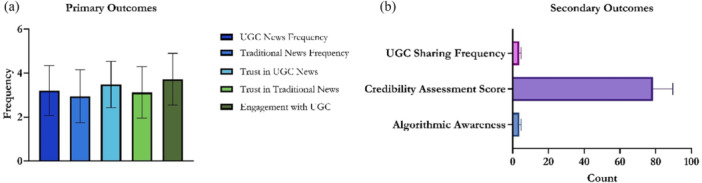
Descriptive statistics for secondary outcomes and primary outcomes. (a) Primary Outcomes: It presents a bar chart showing the descriptive statistics (mean values) for five key metrics related to news consumption habits, trust, and engagement. (b) Secondary Outcomes: It presents a horizontal bar chart showing the count or frequency for three additional metrics: UGC sharing frequency, credibility assessment score, and algorithmic awareness.

Participants reported higher UGC news frequency (3.21  ±  1.14) than traditional news (2.95  ±  1.20), with trust in UGC (3.48  ±  1.05) also surpassing trust in traditional sources (3.12  ±  1.17). UGC engagement was highest at 3.72  ±  1.18 (Figure 4a).

Participants reported moderate to high algorithmic awareness (3.75 ± 1.07, median = 4.0), indicating recognition of the influence of recommendation systems (Figure 4b). Credibility assessment averaged 78.4  ±  11.2 (median = 80.0, range = 50–100), while UGC sharing frequency was 3.65  ±  1.10 (median = 4.0), reflecting active redistribution of user‐generated news.

Trust in UGC varied by country, reflecting underlying legal frameworks and media dependency norms. In Brazil and China, higher trust is correlated with weaker institutional media trust and limited regulation of misinformation, aligning with media dependency theory. U.S. and Japanese participants demonstrated selective trust, influenced by stricter legal environments and higher algorithmic awareness, consistent with UGT. Indian respondents exhibited high engagement but lower content verification, suggesting that social validation shaped trust more than formal policy. These findings highlight how regulatory ecosystems and cultural norms influence perceptions of UGC trust.

ANOVA revealed significant cross‐country differences in UGC news frequency [*F*(4, 95) = 4.32, *p* = 0.003] and trust in UGC [*F*(4, 95) = 3.75, *p* = 0.007], but not in traditional news trust [*F*(4, 95) = 1.89, *p* = 0.112] (Table 1). U.S. and Indian participants reported higher UGC trust and engagement, reflecting UGT, where users actively seek participatory content. In contrast, lower trust in China and Japan aligns with Media Dependency Theory, as state‐regulated environments shape reliance on institutional sources of information.

**Table 1:** ANOVA results for differences in primary outcomes across countries.

Outcome variable	*F*‐statistic	*p* value
UGC news frequency	4.32	0.003**
Traditional news frequency	2.98	0.021*
Trust in UGC news	3.75	0.007**
Trust in traditional news	1.89	0.112
Engagement with UGC	5.12	< 0.001**

* *p* < 0.05

** *p* < 0.01.

A multiple regression predicting trust in UGC news showed that algorithmic awareness (*β* = 0.28, *p* < 0.001), credibility assessment (*β* = 0.14, *p* = 0.002) and UGC sharing frequency (*β* = 0.21, *p* < 0.001) were all significant positive predictors (*R*² = 0.41) (Table 2). These results suggest that participants with higher awareness of algorithmic curation, stronger evaluation skills and greater participatory behaviour were more likely to trust UGC. This supports the UGT, which posits that active content sharing reflects trust built through goal‐directed media use, and the media dependency theory suggests that trust is shaped by the user's evaluative reliance on digital infrastructures.

**Table 2:** Multivariate regression predicting trust in UGC news.

Predictor variable	Coefficient (*β*)	SE	*t*‐value	*p* value
Algorithmic awareness	0.28	0.06	4.67	< 0.001**
Credibility assessment score	0.14	0.04	3.82	0.002**
UGC sharing frequency	0.21	0.05	4.15	< 0.001**
Constant	1.92	0.54	3.56	0.001**

*Note: R*² = 0.41,

** *p* < 0.01

**Figure 5:**
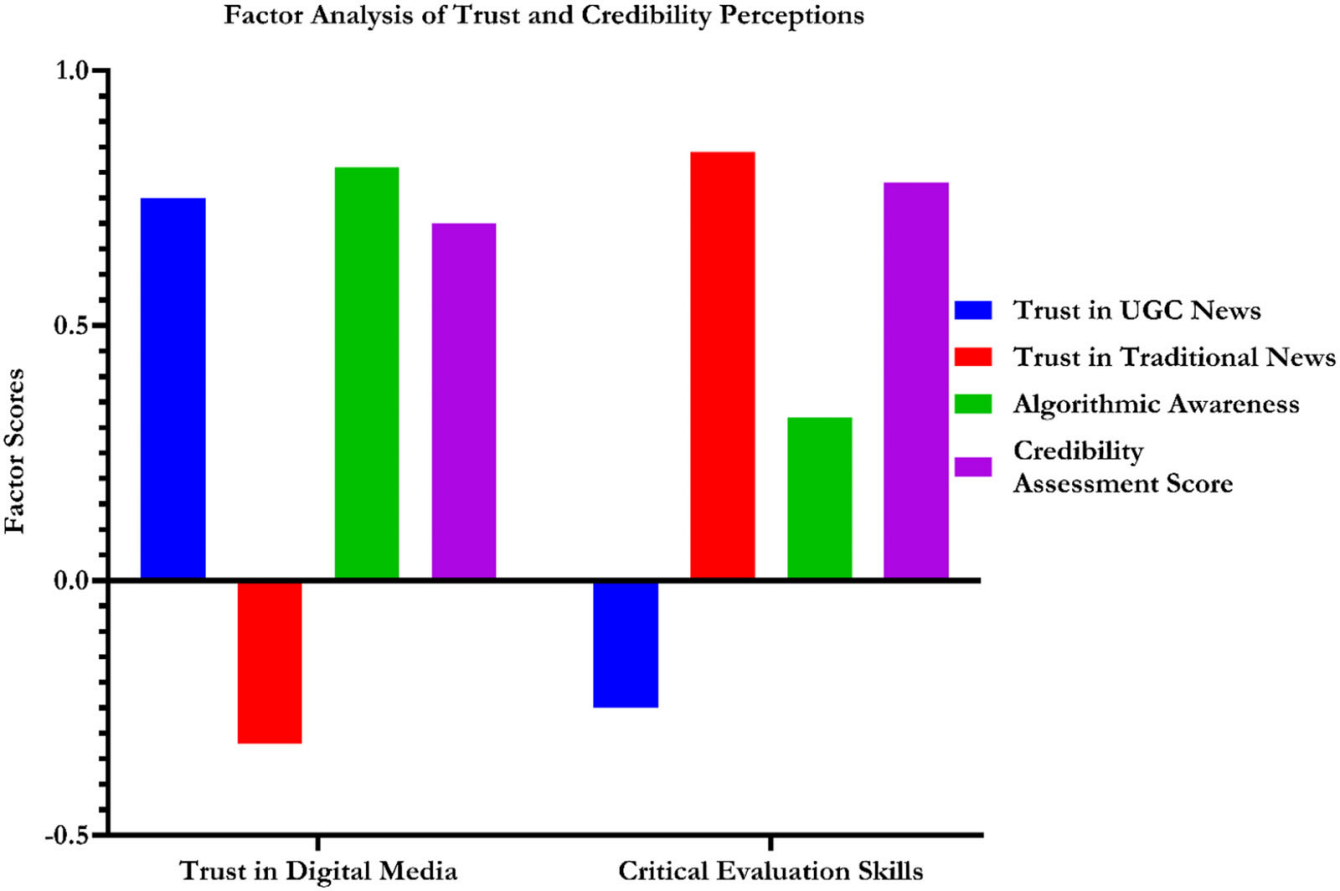
Factor analysis of trust and credibility perceptions.

A factor analysis revealed two latent constructs shaping trust and credibility perceptions (Figure 5). Factor 1 (Trust in Digital Media) exhibited strong loadings for trust in UGC news (0.75), algorithmic awareness (0.81) and credibility assessment (0.70), whereas trust in traditional news loaded negatively (−0.32). This pattern suggests that individuals who trust digital media are more critically aware of algorithmic curation and apply evaluative skills to UGC content. These findings align with media dependency theory, as users increasingly rely on digital systems for credible information, and also support UGT, which posits that discerning users actively engage with content that meets their evaluative needs.

SEM (Table 3) indicated that UGC engagement positively predicted trust in UGC news (*β* = 0.42, *p* < 0.001), while algorithmic influence reduced trust (*β* = –0.33, *p* = 0.015). Credibility perceptions increased engagement (*β* = 0.27, *p* = 0.028), and trust in UGC further enhanced viewer engagement (*β* = 0.31, *p* = 0.010). Conversely, algorithmic influence was associated with diminished engagement (*β* = –0.18, *p* = 0.032). Content verification strongly predicted trust (*β* = 0.37, *p* < 0.001). These dynamics align with UGT, suggesting that users engage more when content meets their expectations of trust and credibility.

**Table 3:** Structural equation modelling (SEM) results based on direct effects.

Path	Standardised coefficient (*β*)	SE	95% CI	*p* value
UGC engagement → Trust	0.42	0.08	[0.28, 0.56]	< 0.001**
Algorithm influence → Trust	−0.33	0.07	[−0.47, −0.19]	0.015*
Credibility → Engagement	0.27	0.06	[0.15, 0.39]	0.028*
Trust → Viewer engagement	0.31	0.07	[0.17, 0.45]	0.010*
Algorithm influence → Viewer engagement	−0.18	0.05	[−0.29, −0.07]	0.032*
Content verification strategy → Trust	0.37	0.08	[0.22, 0.52]	< 0.001**

* *p* < 0.05

** *p* < 0.01.

SEM results confirmed that trust in UGC news partially mediated the link between UGC engagement and viewer engagement (Table 4). The indirect effect was significant (*β* = 0.13, *p* = 0.023), reinforcing trust as a mechanism driving participation. Algorithmic influence reduced engagement by negatively impacting trust (*β* = –0.10, *p* = 0.038), whereas content verification increased engagement by enhancing trust (*β* = 0.12, *p* = 0.041). These findings support UGT, illustrating how trust and verification fulfil cognitive needs that enhance UGC engagement despite algorithmic filtering.

**Table 4:** Structural equation modelling (SEM) results based on indirect and total effects.

Path	Indirect effect	SE	*p* value	Total effect	SE	*p* value
UGC engagement → Viewer engagement (via trust)	0.13	0.05	0.023*	0.56	0.09	< 0.001**
Algorithm influence → Viewer engagement (via trust)	−0.10	0.04	0.038*	−0.28	0.07	0.021*
Content verification → Viewer engagement (via trust)	0.12	0.06	0.041*	0.44	0.08	< 0.001**

* *p* < 0.05

** *p* < 0.01.

Moderation analysis showed that algorithm awareness significantly shaped how users responded to algorithmic influence (Table 5). Awareness reduced trust in UGC when content was algorithmically recommended (*β* = −0.22, *p* = 0.018) and lowered engagement with such content (*β* = −0.30, *p* < 0.001). However, awareness increased trust when users actively engaged with UGC (*β* = 0.15, *p* = 0.045). These results align with cognitive dissonance theory, suggesting that users aware of algorithmic bias may experience dissonance when consuming curated content, which lowers trust unless engagement is voluntary; in this case, trust is reaffirmed through active, self‐directed consumption.

**Table 5:** Moderation analysis of effects of algorithm awareness on trust and engagement.

Moderator variable	Path	Interaction effect (*β*)	SE	*p* value
Algorithm awareness	Algorithm influence → Trust	−0.22	0.07	0.018*
Algorithm awareness	Algorithm influence → Viewer engagement	−0.30	0.06	< 0.001**
Algorithm awareness	UGC engagement → Trust	0.15	0.05	0.045*

*Note:* Significant interaction effects suggest that higher algorithm awareness weakens the negative impact of algorithmic influence on trust and engagement.

* *p* < 0.05

** *p* < 0.01.

Control variables in the SEM model (Table 6) showed that education and UGC sharing significantly predicted trust and engagement. Higher education was correlated with increased trust (*β* = 0.16, *p* = 0.015) and engagement (*β* = 0.18, *p* = 0.015). In contrast, frequent UGC sharers showed robust effects on both outcomes, with trust being most pronounced (*β* = 0.27) and engagement also significant (*β* = 0.22); both *p* < 0.001. Gender influenced engagement more than trust, while age had no significant impact. These findings align with Hofstede's cultural dimensions, particularly individualism and uncertainty avoidance, suggesting that educated, digitally active users in low‐avoidance cultures are more trusting and participatory in UGC news.

**Table 6:** Control variables in SEM model.

Control variable	Trust in UGC news (*β*)	Viewer engagement (*β*)	*p* value
Age	−0.12	−0.09	0.061
Gender (female)	0.10	0.14	0.042*
Education level	0.16	0.18	0.015*
UGC sharing frequency	0.27	0.22	< 0.001**

*Note:* Results indicate that education level and UGC sharing behaviour significantly impact trust and engagement with UGC news.

* *p* < 0.05

** *p* < 0.01.

**Figure 6:**
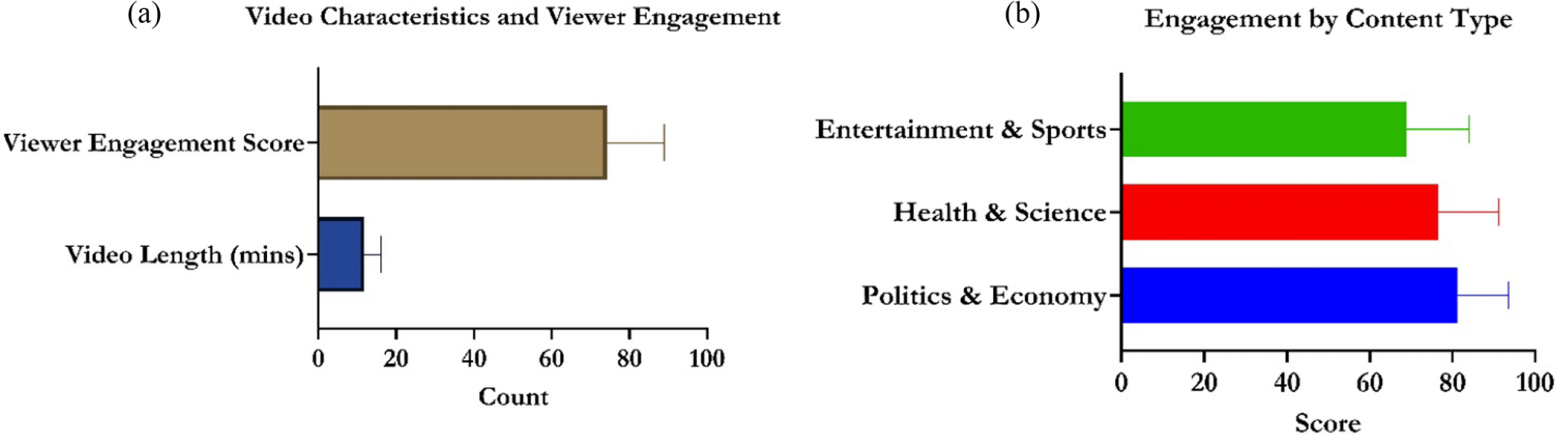
Content analysis based on video characteristics and viewer engagement. (a) Video Characteristics and Viewer Engagement，shows the relationship between video length (in minutes) and viewer engagement score. (b) Engagement by Content Type，shows the viewer engagement scores for different types of news content (Entertainment & Sports, Health & Science, Politics & Economy).

The content analysis of 200 YouTube news videos revealed an average length of 11.8 ± 4.3 min, balancing depth and viewer retention (Figure 6a). Viewer engagement averaged 74.3 ± 14.6, with wide variation by content type. Hard news (e.g., politics, economics) scored highest (81.2 ± 12.3), comprising 35% of the sample (Figure 6b), followed by health/science news (76.5 ± 14.7, 25%). Soft news (e.g., entertainment, sports) had the lowest engagement (68.9% ± 15.2%), but made up 40% of the videos. These patterns suggest that fact‐based, analytical content elicits stronger interaction than entertainment‐focused UGC news.

**Figure 7:**
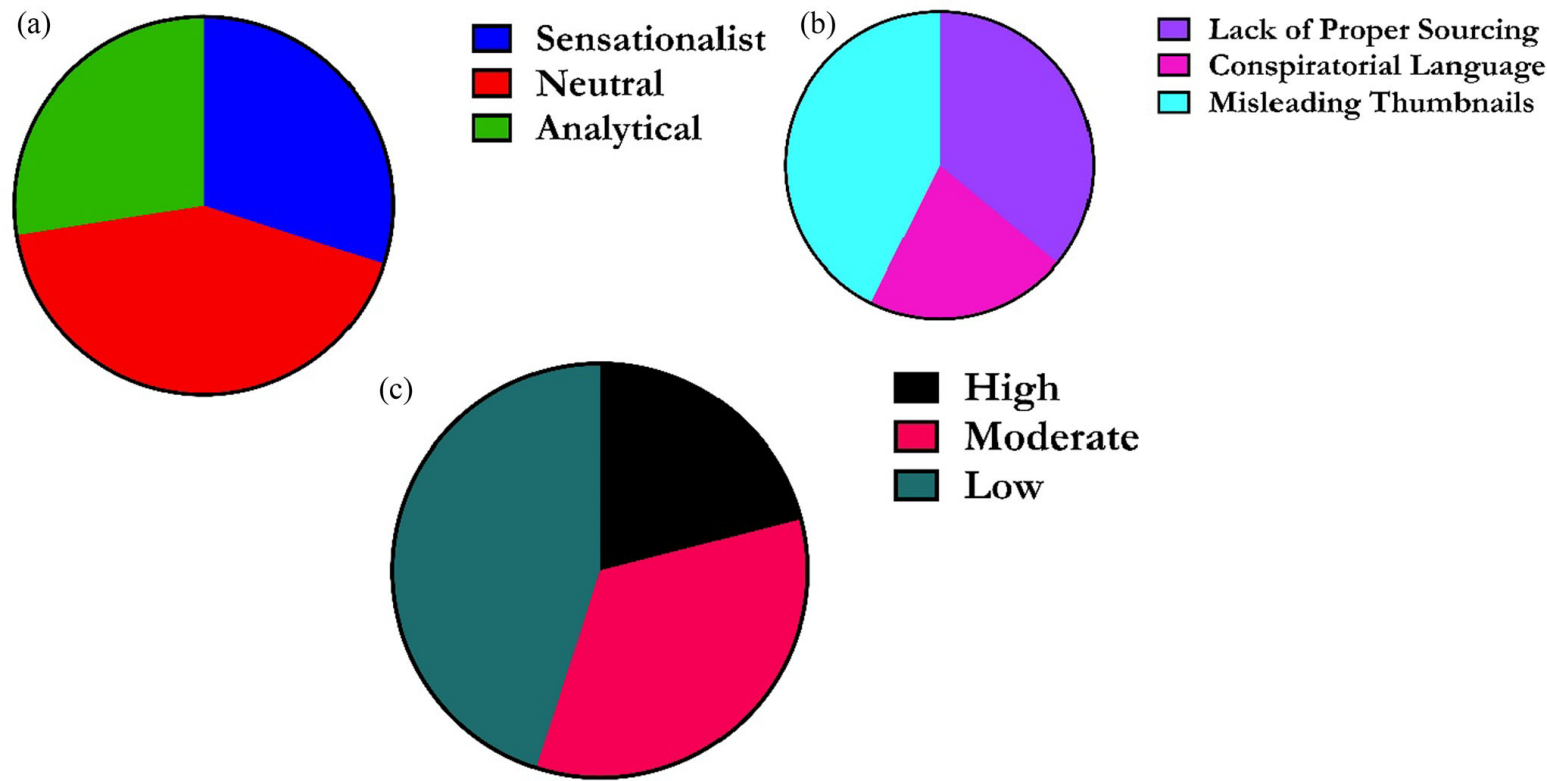
Content analysis of news framing, source credibility and patterns of misinformation. (a) It shows the distribution of different types of news framing found in the analysis. The categories are Sensationalist, Neutral, and Analytical. (b) It shows the prevalence of different patterns of misinformation or low‐credibility tactics. The categories are Lack of Proper Sourcing, Conspiratorial Language, and Misleading Thumbnails. (c) It shows the distribution of source credibility ratings for the videos. The categories are High, Moderate, and Low.

Content analysis (Figure 7) revealed that 45% of YouTube news videos were of low credibility, with only 21% rated as highly credible. While neutral framing dominated (42.5%), sensationalist content (30%) drew significantly higher engagement despite lower credibility. This pattern aligns with UGT, where users are drawn to emotionally charged content for affective gratification, even at the expense of accuracy. Videos with misleading thumbnails used in 45% of cases achieved 15% more engagement, reflecting the persuasive power of visual sensationalism. These findings reveal how misinformation tactics exploit attention‐driven algorithms, underscoring the urgent need for credibility‐based curation and improved digital media literacy.

The sentiment analysis of user comments (Table 7) showed that 40% were positive, often commending UGC news for clarity and insight. Neutral responses (35%) reflected cautious engagement, with many seeking additional sources or clarification. Negative comments (25%) challenged the credibility of the content, labelling it as biased or misleading. These patterns illustrate a mixed trust environment, where viewers oscillate between appreciation and scepticism, consistent with Media Dependency Theory, which posits that trust in media is shaped by perceived content reliability and structural media dynamics in the user's information environment.

**Table 7:** Content analysis of comment sentiment and misinformation patterns.

Sentiment category	% of comments	Example sentiments
Positive	40.0%	‘Great analysis! Informative.’
Neutral	35.0%	‘Interesting perspective, but needs more sources.’
Negative	25.0%	‘Fake news, misleading content!’

Trust in UGC news varies by cultural orientation. U.S. and Indian participants showed strong trust when creators demonstrated transparency, while Chinese users relied more on institutional authority, aligning with Hofstede's power distance and long‐term orientation (Table 8). Japanese users demonstrated high institutional trust but lower reliance on individual creators, highlighting the cultural value placed on source credibility and social order.

**Table 8:** . Hofstede cross‐cultural trust and credibility perceptions in UGC news.

Country	Institutional trust level	Creator transparency impact	Fact‐checking behaviour	Dominant cultural traits (Hofstede)
United States	Medium–high	Strongly increases trust	Frequently cross‐verifies	Low power distance, high individualism, low uncertainty avoidance
Brazil	Medium	Moderately increases trust	Occasionally verifies	High uncertainty avoidance, high collectivism
India	Medium	Significantly increases trust	Often triangulates sources	High power distance, high collectivism
China	Low–medium	Limited trust improvement	Rarely verifies	High power distance, high long‐term orientation
Japan	High institutional trust	Mildly increases trust	Relies on official sources	High uncertainty avoidance, high masculinity

Engagement and ethical expectations reflect national values. U.S. and Japanese audiences demand transparency and accuracy, showing low tolerance for sensationalism (Table 9). Brazilian and Indian users value authenticity and the context of community. Chinese participants exhibited passive consumption and relied on state‐affiliated cues, reflecting a high tolerance for sensationalism shaped by media regulation and cultural acceptance of authority in news environments.

**Table 9:** Cross‐cultural differences in UGC engagement, trust moderation and ethical expectations.

Country	Engagement style	Algorithmic scepticism	Sensationalism tolerance	Ethical expectations from creators
United States	Interactive, comment‐heavy	High	Low	Emphasis on disclosure and fact‐checking
Brazil	Share‐driven, emotional	Medium	Medium–high	Balanced: authenticity over formal accuracy
India	Forwarding‐based, group‐led	Medium–high	Moderate	High value placed on informative intent
China	Passive viewing	Low	High	Censorship compliance and brand credibility
Japan	Selective, subscription‐based	High	Low	Precision, moderation and institutional tone

## 7 Discussion

This study demonstrates that young users engage with UGC not only for access, but also to fulfil active informational and participatory needs, thereby validating core tenets of UGT. Trust in UGC was not uniformly granted; it emerged through selective sharing, credibility checks and resistance to algorithmic manipulation. Participants reported greater confidence in creators who disclosed sources, suggesting that gratification stemmed from perceived transparency and personal relevance. While dos Santos Catalão [15] emphasises UGC's role in democratising real‐time news access, our findings extend this by showing that gratification is increasingly shaped by users' ability to evaluate content amid algorithmic saturation. Sensationalism attracted clicks but failed to establish sustainable trust, underscoring that engagement metrics alone do not meet users' deeper cognitive needs. These dynamics underscore that UGC consumption is a purposive behaviour, driven by youth expectations for autonomy, credibility and control, especially in contexts where institutional media are viewed as opaque or less relatable. Thus, our findings indicate that enhancing media literacy can serve cognitive needs and contribute to healthier information environments, especially by equipping young people to discern between trustworthy and misleading content in ways that support informed health decision‐making.

This study confirms that UGC on YouTube has become a primary news source for many young users, surpassing traditional journalism in perceived trust and engagement. This is particularly important for public health, where false or misleading health information can erode confidence in evidence‐based practices. Embedding digital media literacy in health communication frameworks can help counteract the risks of algorithmically amplified misinformation. Trust was strongly influenced by transparency, consistency and perceived authenticity of independent creators [1, 17, 27, 32]. However, algorithmic personalisation emerged as a double‐edged force. Participants with higher algorithmic awareness were more sceptical of platform‐suggested content, often counteracting recommendation biases through fact‐checking and source triangulation. This behaviour reflects the core of algorithmic gatekeeping theory, which posits that algorithmic systems, not just human editors, act as gatekeepers, shaping exposure, credibility perceptions and engagement. Our findings extend previous studies [15, 22] by demonstrating that while UGC democratises participation, algorithmic filters prioritise engagement over accuracy. This curation bias amplifies sensationalist framing and underlines the urgent need for platforms to recalibrate algorithms toward credibility rather than click‐through incentives.

The evolving landscape of digital journalism continues to show that trust in content creators remains intact despite the decentralisation of news. Given the rise of persuasive storytelling that mimics health news, the role of media dependency in UGC consumption must be addressed through media literacy programs embedded in health education curricula, particularly for populations reliant on digital content for health knowledge. Prior work has shown how YouTubers shape adolescent identity and peer‐level information exchange [3, 4, 36]. Our findings build on this by showing that UGC credibility is tightly linked to transparency, investigative depth and creator–audience interaction. However, persuasive or entertainment‐focused content often blurs journalism with opinion, increasing the risk of misinterpretation. Drawing from media dependency theory, our results illustrate that young users, especially in media‐saturated environments, develop trust based on their dependency on specific digital platforms for information. In contexts where institutional journalism is less accessible or less trusted, users become more reliant on UGC creators, amplifying their susceptibility to persuasive storytelling and implicit bias. This aligns with [14, 41], who found that UGC is often perceived as more trustworthy than overt advertising. Such dependency structures underscore the urgent need for media literacy training that helps youth distinguish entertainment from credible journalism

Platform responsibility and content authentication are central concerns due to widespread misinformation in UGC‐driven news. In the context of health misinformation, culturally responsive media literacy strategies are essential to ensure vulnerable populations are not misled by unverified user‐generated health claims, particularly in contexts where traditional health communication systems are less dominant. Youth are particularly vulnerable, as youth‐targeted UGC often lacks robust fact‐checking or regulatory safeguards [28]. Our findings extend this by showing that misleading thumbnails, conspiratorial framing and lack of citations correlate with higher engagement, especially when sensationalism dominates. As Xiang (2019) noted, UGC reshapes citizen journalism by shifting agenda‐setting to digital communities. However, this empowerment varies by culture. In Hofstede's cultural dimensions [42, 43, 44], countries high in individualism (e.g., the United States, Brazil) show greater reliance on peer‐generated content and self‐verification, contributing to fragmented trust structures. In contrast, participants from high uncertainty avoidance cultures (e.g., Japan, China) demonstrated lower tolerance for unverified content and higher demands for institutional control. These cultural dynamics help explain varied attitudes toward platform accountability, emphasising the need for localised content moderation strategies that align with cultural expectations for information integrity and risk management

This study deepens understanding of how engagement shapes trust and credibility in UGC‐driven news. This intersection between digital trust, cultural expectations and UGC underscores the urgent need for public health institutions to invest in culturally sensitive media literacy education. Such programs can strengthen digital resilience and ensure that health‐related engagement online supports accurate information uptake. While UGC enhances participatory journalism [15, 16, 22], our findings show that trust depends on creator credibility, algorithmic exposure and misinformation susceptibility. Cultural context significantly moderated these effects. Participants in high power distance cultures (e.g., China) showed greater trust in institutional sources and were more sceptical of decentralised UGC. Conversely, low power distance cultures (e.g., the U.S.) favoured peer content and valued creator transparency. Participants from long‐term oriented societies (e.g., Japan) were more cautious with algorithmically curated content, reflecting a cultural preference for accuracy and reliability over immediacy. These patterns reveal that digital literacy cannot be separated from cultural context. Thus, platform governance should tailor content verification and algorithmic transparency to national norms. Media literacy efforts must also account for culturally embedded attitudes toward authority, credibility and information responsibility in digital spaces.

## 8 Theoretical and Practical Implications

This study advances theory by integrating UGT, MRT and Algorithmic Gatekeeping to explain how engagement, credibility and algorithmic exposure shape UGC news trust. It highlights trust as a mediating mechanism and algorithmic awareness as a moderator, extending prior models. Practically, the findings call for algorithmic transparency, improved content verification and tailored media literacy programs. Platforms should prioritise credible content over engagement‐maximising sensationalism, while policies must support user empowerment in navigating digital news environments critically and responsibly. These findings also inform public health communication, where digital media literacy is central to combating misinformation in crisis contexts. Integrating critical literacy into public health policy can reduce vulnerability to misleading health‐related UGC.

## 9 Limitations and Future Research

This study's limited sample size across five countries may not capture global variations in UGC news consumption, digital literacy, or regulatory contexts. Broader, multi‐regional sampling is needed to inform location‐specific governance and misinformation policies. The cross‐sectional design prevents causal inferences; thus, future longitudinal research should examine evolving trust patterns in UGC engagement. Experimental designs are also recommended to assess how different misinformation interventions affect user trust and interaction with digital news sources. Future studies should also explore how digital literacy mediates the impact of UGC exposure on public health information trust across socio‐cultural contexts.

## 10 Conclusion

This study affirms that UGC is rapidly becoming the dominant mode of news consumption, trust formation and engagement among young audiences. Trust in UGC exceeded that in traditional news, shaped by creator transparency, algorithmic exposure and misinformation vulnerability. While algorithmic awareness tempered bias, sensationalist content, despite low credibility, drew higher engagement. These findings underscore UGC's dual role as both democratising and ethically problematic. Strengthening real‐time verification, improving source labelling and enforcing platform moderation are critical. Media literacy must evolve to equip youth with skills to discern fact‐based journalism from entertainment, ensuring that digital news ecosystems remain credible and accountable. Strengthening media literacy is a digital competency and a public health imperative, ensuring communities are resilient to health misinformation proliferated through UGC platforms.

## 11 Ethical Considerations

All participants provided informed consent before participating in the study. For participants aged 13–17, parental or guardian consent was obtained in addition to their assent. The study adhered to ethical research principles outlined by the China Southwest Medical University Institutional Review Board (No. SWM‐0098‐DS4), which approved the research protocol. Confidentiality and anonymity of all respondents were maintained throughout the study, with data securely stored and used solely for research purposes. Participants had the right to withdraw at any stage without consequence.

## Author Contributions

**Wu Chunqiong:** conceptualization (lead), methodology (lead), formal analysis (lead), investigation (lead), resources (lead), writing – original draft (lead), writing – review & editing (lead), visualization (lead), supervision (lead), project administration (lead). **Jiang Shan:** writing – review & editing (supporting). **Sun Jianhong:** writing – review & editing (supporting). **Liu Yingqi:** writing – review & editing (supporting).

## Ethics Statement

This study was approved by the Institutional Review Board of China Southwest Medical University (Approval No. SWM‐0098‐DS4). All research procedures adhered to ethical guidelines for human subjects' research.

## Consent

All participants provided informed consent before taking part in the study. For participants aged 13–17, parental or guardian consent was obtained in addition to participant assent.

## Conflicts of Interest

The authors declare no conflicts of interest.

## Data Availability

The data supporting this study's findings are available upon reasonable request from the corresponding author. Due to ethical considerations and participant confidentiality, certain data may be restricted. All data supporting the findings of this study are fully available within the article.
